# Hospital and Institutional News

**Published:** 1915-07-03

**Authors:** 


### HOSPITAL AND INSTITUTIONAL NEWS.

### SOLDIERS AND WARD DISCIPLINE.

We have already drawn attention to the problems of discipline in the new military hospitals which necessarily arise from the fact that the original staff, when given commissions, are not thereby endowed with a knowledge of military procedure, while the soldiers, accustomed to this discipline, are tempted to take advantage of the inexperience of their officers. An instance of the problem which may occur in a voluntary hospital where certain beds are set apart for the wounded was briefly referred to at a meeting of the executive committee of Worcester General Infirmary last week. A report was presented by the house committee to the effect that a complaint was received from the wounded soldiers "against the action of the matron and sisters." Two of the soldiers appeared before the committee, who heard their complaint, and the Chairman informed them that ward discipline must be maintained, and that the regulations must be obeyed. As the nature of the complaint is not apparent, comment would be fruitless; but we record the incident as showing how much self-restraint is required by all parties in the process of adapting the voluntary hospitals and military patients to each other. It is very gratifying to think that, notwithstanding the modification of procedure involved in ordinary hospital management, friction has been, on the whole, so infrequent, and as time goes on there will be less occasion for it, when the new conditions become a familiar routine to the staffs.

### GOVERNORS' VOTES AND GOVERNORS' GUINEAS.

A remarkable discussion took place last week at the annual meeting of the Great Yarmouth Hospital concerning a suggested amendment of the rules. The Rev. R. R. Young moved an amendment to them, under which in future all governors and subscribers to the hospital of half-a-guinea and upwards would have one vote each at governors' and subscribers' meetings. At present, he pointed out, each subscriber is entitled to one vote for every guinea subscribed up to a maximum of eight votes. He described this as an antiquated arrangement, since the interest of any lady or gentleman could not be measured by the length of their purses. By enlarging the constituency, he added, the meetings would be better attended and more interest would be taken in the hospital's work. In the discussion which followed, the Mayor, Alderman McCowan, supported this contention, and said that because a man was rich enough to subscribe twenty guineas, therefore he should have twenty votes, was not the principle acted on in these days. On the other hand, it was argued that the subscribers who had multiple votes often represented a large number of small subscribers; and, further, that if the friendly societies found their voting power reduced subscriptions to the hospital would suffer. In the end it was virtually decided to leave matters where they were by carrying an amendment to the effect that subscribers should be entitled to one vote for each half-guinea up to a maximum of eight votes. Thus the qualification for a vote has been reduced from one guinea to half-a-guinea, but the principle of multiple votes has been retained. We think and hope that the final stage of one man one vote, irrespective of the size of any subscription beyond the qualifying figure, will come in time, but meanwhile Mr. Young is to be congratulated on having raised the matter. In our view it is more important to limit each subscriber to one vote than to reduce the subscription qualification below one guinea.

### GIFT OF TWENTY-FIVE BEDS TO A DUBLIN HOSPITAL.

Among the many generous gifts which the voluntary hospitals have experienced in connection with their work for the wounded mention must be made of a remarkable example of generosity which was gratefully acknowledged at the June meeting of the board of the Royal City of Dublin Hospital. Details of the gift were contained in a letter from the County Dublin branch of the British Red Cross Society, which stated that Viscount Iveagh had generously offered to provide and equip at his own expense twenty-five additional beds for the use of the sick and wounded in the hospital. A resolution of thanks was passed, and the Secretary announced that these beds were now erected and would be fully equipped within a few days. We may therefore take it that this important addition to the hospital's accommodation is by this time in working order, and we note with pleasure that an appeal in the newspapers for Merlin chairs for the wounded had produced five chairs and a gift of £5 for the purchase of another. These timely gifts should further consolidate the support of the hospital's well-wishers, who, we trust, will not forget that, in addition to incidental requirements of the above character, special help is required to pay off the overdraft, which now amounts to some £6,500.

### AUSTRALIAN HOSPITALS AND THE WAR.

All our hospitals at present are short of funds, owing to the great outflow of money in aid of Belgium and the Red Cross work [writes our Australian correspondent]. It is reported that the Western Suburbs Cottage Hospital (Sydney) is on the point of closing for this reason. The Adelaide Hospital, which is many years out of date, has no money to build necessary additions, and is also at present overcrowded. Sometimes there are 340 patients to 270 beds—the surplus having to sleep on the floor. The Melbourne Hospital is again in debt as to its maintenance fund, owing to decreased subscriptions. The Federal Government, in addition to the hospitals already sent abroad with the first expeditionary troops, has decided to offer to the Mother-country a double general hospital for service. It will contain 1,040 beds, with 35 doctors and 146 nurses, while 250 N.C.O.s and men will perform the duties of orderlies and bearers. As far as possible, all equipment, medical stores, instruments, etc., will be supplied by the Commonwealth. All the hospitals are also short of medical officers; 21 medical men left the Melbourne Hospital alone for the war, and the other institutions are affected in proportion. The 100 doctors requested for the Royal Army Medical Corps have also been sent, the last batch having sailed a fortnight ago, at the time of writing.

### THE NEW RESEARCH INSTITUTE AT MELBOURNE.

An institute or department of pathological research has long been a need in Melbourne. It was known that the plans of the new Melbourne Hospital provided for a pathological block, and by March that it was in course of being erected. Thereupon the trustees of the "Walter and Eliza Hall Fund" stepped forward with the offer of a subsidy of £2,500 per annum towards an Institute of Medical and Pathological Research. The hospital has accepted the offer, which necessitates the building being made one storey higher. The institute is to be administered by a board consisting of the president of Melbourne Hospital Committee (to act as president of the board), two representatives appointed by the Hall Fund (one of whom to act as vice-president), the Vice-Chancellor of the Melbourne University, the professor of pathology at the University, and one representative chosen yearly by the Melbourne Hospital medical staff. The secretary of the Melbourne Hospital will act as secretary of the board. The trustees reserve the right to terminate the subsidy if at any time the work is not deemed satisfactory or if the hospital should pass under direct Government control. It is recommended, but not prescribed, that the director of the institute should receive a salary of £800 per annum (£500, as at present, to be derived from clinical fees from students); one first assistant, £400; two assistants, £250; two laboratory assistants, £150; material, apparatus, etc., £200; library, etc., £125; clerical assistance, £125; upkeep, etc., £175. A balance is provided for contingencies, and it is suggested that £75 per annum should be invested by way of a retiring allowance of £375 for the director, who thus, obviously, is meant to hold office for five years only. One year's payment is to be advanced as for the year April 1, 1914, to March 31, 1915, and in October half-yearly payments of £1,250 will commence. It should be remembered that the late Mr. Walter Hall's widow set apart £1,000,000 for public purposes.

### THE TRANSFORMATION OF LIVINGSTONE COLLEGE.

With reference to the paragraph which recently appeared in our columns referring to the possible closing of Livingstone College, Leyton, we now learn that this is an accomplished fact, and that the college has been taken over as a supplementary institution to the Bethnal Green Military Hospital. The building will accommodate some forty-five beds, and, it is stated, will be opened early in August for the reception of surgical cases.

### THE PUBLIC AND THE AMBULANCE SERVICE.

A feature essential to the success of the London ambulance system is the use of private telephones by police constables in uniform, for the purpose of calling the ambulance. An indicating tablet is placed upon premises where telephones are, by the courtesy of the occupants, available for the purpose. By publicity through the Press and by appeals to the Borough Councils some 350 telephones are at present available in various parts of London. A large proportion of these are in premises in occupation of the County Council or the Borough Councils, so that the number of telephones placed at the disposal of the service by private telephone subscribers is extremely small. The Council are now in communication with the large business firms who have branches in several districts of the county, and they expect by this means to secure a large addition to the number of telephones available. To secure efficiency, telephonic facilities should be available at intervals of not more than 440 yards in every one of the more important streets and roads in the county. We hope that the Council's appeal for help will not fall upon deaf ears, for once that body decided that it was impossible to have special telephone posts for its ambulance service, the voluntary assistance of the public became imperative. Every telephone thus obtained means the quicker arrival of the ambulance and the speedier removal to hospital.

### INDIAN APPLICANTS FOR MEDICAL POSTS.

According to report the Rochdale Health Committee have had a curious experience in connection with their attempt to fill a vacancy on their medical staff. It is said that a salary of £400 was offered, but that only five candidates appeared, only one of whom was English—namely, a lady. Three Indians and one Belgian were her fellow-applicants, and eventually the Belgian, who since the war is understood to have had a post at Halifax, was appointed. This not only shows the scarcity of British doctors, but raises questions as to the extent of the opportunity now open to medical men of Indian birth. Such men have usually sought a medical degree in order to return to practise in the country of their birth, and it would be interesting to know if the experience of the Rochdale Health Committee is shared by other authorities.

### THE ANNUAL FESTIVAL OF THE ORDER OF ST. JOHN.

On St. John the Baptist's Day, last week, the annual festival of the Order of St. John of Jerusalem in England was celebrated at the commemoration service in St. John's Church, Clerkenwell. The roll of the departed members of the Order and of the St. John Ambulance Brigade was read. During the war the number of members who have lost their lives is forty-six. The General Assembly of the Order was also held in the Chapter Hall at St. John's Gate. The St. John Ambulance Association, which celebrates its thirty-eighth birthday this year, has provided some 8,000 men and 400 nurses for the present war, which is sufficient to show the valuable results achieved through the spread of the Order's branches all over the country.

### THE ROOT DIFFICULTY IN INSURANCE SANATORIA.

THE difficulties inherent in the carrying out of any adequate scheme for the treatment of tuberculous persons in a given area were made apparent at a meeting of the West Riding Insurance Committee held last week. Considerable discussion took place on a recommendation of the Sanatorium and Administrative Sub-Committee that the granting of sanatorium benefit to the dependants of insured persons should shortly be suspended. The point of view of the Insurance Committee was not any objection to the treatment of dependants, but that the responsibility for such cases rested with the County Council, and that as the income of the Insurance Committee was derived from the contributions of insured persons who paid for the sanatorium benefit, it was not just that any of their contributions should be transferred to any other channel. It was stated that insured persons had been kept waiting for vacancies in sanatoria, or had been turned out prematurely in order to make room for dependants. Although apparently no one present at the meeting refuted the latter allegation it is extremely improbable that there is any foundation for it. One cannot imagine a medical superintendent being influenced by any other than clinical reasons for discharging a patient who is not insubordinate. The root of all difficulties with schemes for the treatment of tuberculous persons is lack of accommodation, and this deficiency can only gradually (and now slowly) be made good. It is obviously very unsatisfactory not to be able to offer treatment to the dependants of insured persons, for thus some of the insured persons themselves will run a greater risk of acquiring tuberculosis and of becoming a burden in turn. But, naturally, the insured persons consider they have a prior claim to any sanatorium accommodation that is available.

### COUNTY COUNCILS AND INSURANCE COMMITTEES.

COUNTY Councils are not prepared, as may easily be understood, to provide, especially at the present time, more sanatoria unless funds are forthcoming from national sources with a guarantee for several years. In the case of the West Riding Insurance Committee a good bargain appears to have been made with the County Council, and it would be practically impossible for the Insurance Committee to obtain better value for the money elsewhere. The financial aspect of the case appears to imply that the expense of the dependants was borne by the Government grants and the rates, for the contributions of the Insurance Committee almost equalled the cost of the insured persons. The fact of the matter is that the properly controlled treatment of tuberculous persons in the kingdom is a bigger proposition than many people imagine, and a much more expensive one too. Not a few authorities, both lay and medical, are apprehensive as to whether the money is being well spent, and certainly it has been urged that tuberculosis is not likely to be diminished any more rapidly with our present efforts than without them. The present is decidedly an inopportune time to embark on vaster measures.

### BOARD-ROOM PHOTOGRAPHS.

OUR suggestion that memorial tablets should be placed in the institutions whose former secretaries have been killed on active service has already been adopted in the case of the Hampstead General Hospital, and will, we hope, be also acted on at East Sussex Hospital, Hastings. A proposal, however, to commemorate the gallant deeds of those members of the staff who have achieved distinction for war services by hanging their photographs in the board-room is being acted on at the Royal Infirmary, Sheffield. At the quarterly meeting last week Mr. H. H. Bedford announced the arrival of a photograph of Lieutenant T. Clarke, who had been mentioned in dispatches for bravery in the field. He said that this photograph was to be framed and hung, with one of Dr. Gwynne, in the board-room. Both medical men have been at the infirmary, and such photographs may possess a future interest. In view, however, of the numbers of men connected with certain of the larger institutions now on active service, it might not prove convenient to adopt this practice universally.

### PARENTS AND SCHOOL MEDICAL INSPECTION.

WE publish elsewhere in this issue a second article on "The Educative Work of the School Doctor," in which the question of lantern lectures for parents is considered. Incidents are related from the personal experience of the writer in which these lectures have proved invaluable in breaking down parental prejudice against school medical inspection, and a fascinating picture is given of the relation between parents, children, and school doctor which can be created by a medical man who is keen on his work and prepared to take trouble in making the parents actively interested in it. By way of contrast, the difficulties in the way of this work lately revealed in a Wiltshire police-court are worth mention. The Education Committee of the Wilts County Council summoned six parents for keeping their children away from the annual medical inspection required by the bye-laws. The excuses of the parents for keeping their children away were various. One pleaded sickness on the part of her child, another said the child was kept away because the mother was ill; another stated that he understood that the inspection was not compulsory; another objected to the examination on the ground that the children were frightened. The last pleaded that insufficient warning was given, and on the head master and mistress stating that while written forms had been made out when the examination was fixed, only verbal notices were given to the children on the day previous to the examination itself, the Bench dismissed the summonses. With this, and the question of a case being stated, we are not concerned. The facts are sufficient to illustrate, by contrast, the value of harmonious working, the mode of securing which is so delightfully described in the article on page 297.

### THE MODERN GIRL'S EDUCATION.

A REMARKABLE speech was made by Dr. Mary Scharlieb last week at the annual general meeting of the National Society of Day Nurseries. After stating that the war had emphasised the importance of preserving infant life she paused to criticise the education of the modern girl on the following grounds. Their education, she said, did not fit them to become mothers. They were taught "accomplishments" like modern languages and playing the piano. In short, though Dr. Scharlieb did not put it so bluntly, many girls are "educated" to gain a husband merely, with the result that when they succeed in doing so, and in time become mothers, "the simplest matters regarding their children's well-being" are mysteries to them. The effect was that through ignorance children's lives were lost, and Dr. Scharlieb instanced measles as a disease of early life, the danger of which was not realised through the mother's ignorance of the disease. Her own belief was that the recent rise in infant mortality was due, firstly, to measles, though the employment of women was a subsidiary cause. Consequently schools for mothers and day nurseries were never more needed than at this time. A great work had been done already, but the welfare of our infants and children depended upon the mothers being taught the rules of health. Have the parents any answer to offer?

### CONDITIONAL GIFTS OF CASH IN WAR-TIME.

A GOOD example of courageous belief that the resources of charity are far from exhausted is provided by the generous offer of Mr. James Elliman to provide a sum equal to half of the present indebtedness of the King Edward VII. Hospital, Windsor—namely, £3,000—if the remaining £1,500 can be raised during 1915. Any smaller sum that may be collected Mr. Elliman has also offered to double, but we hope that this will not have the effect of inducing the hospital's supporters to relax their endeavours to obtain the larger sum. Offers of this character, under the voluntary system, are of regular occurrence in ordinary times, but this is the first we recall to be made during the progress of hostilities. In so far as it encourages hospital managers to make their genuine needs plain it is of value, and in any case it should do something to dispel the gloomy view of hospital finance which some hospital men are taking, or affecting to take.

### ACCEPTING MEDICAL ASSISTANTS ON TRUST.

THE cases which appear from time to time in the Courts seem to show that many medical men are distinctly careless in taking for granted the qualifications of would-be assistants. At Manchester last week, for instance, a man was committed for trial on the charges of having forged and uttered certain death certificates. After filling up the certificates the accused was said to have subscribed his name with the letters M.B., Ch.B. Glasgow added. The assistant registrar of Glasgow University gave evidence to the effect that he had been unable to find on the register such a person as the one named on the certificates. The Public Prosecutor's representative stated that the accused applied for the post as assistant to a Dr. Jarvie, a medical practitioner, of Stourbridge, representing himself to be a qualified man, and Dr. Jarvie himself said that he took for granted the qualifications mentioned in the accused's letter of application. As stated, the accused was committed for trial, and whatever the result of that trial may be, and on it, of course, we are precluded from making any comment, the fact remains that medical men are to be found who will take the qualifications of strangers for granted, notwithstanding the ease with which they can be verified by consulting the Medical Register or Directory. We regret that there is sufficient evidence to show that some members of the profession make a habit of taking the professed qualifications of strangers on trust, although it is clear that to do so is not merely, as a layman would say, most unbusinesslike, but also, when the stranger is to occupy the post of assistant, a failure in duty to the patients of the engaging practitioner. To be happy-go-lucky is supposed to be an amiable weakness; where patients are concerned it amounts to a dereliction of duty.

### CHILDREN AND A PUBLIC HEALTH COMMITTEE.

THE Public Health Committee of the Battersea Borough Council has recommended the Council not to subscribe to the funds of the Victoria Hospital for Children, Chelsea. Last year the Council contributed £21 to the funds of the hospital, from which many poor children from Battersea derive benefit, indeed it is the children's hospital nearest to the thickly populated district chiefly inhabited by the very poor. It was felt that the committee had acted unwisely in not acceding to the request for a donation, and they have decided to reconsider the matter, when it is to be hoped they will more generously recognise the claims of the hospital. It is extraordinary that public authorities should propose to withdraw their donations from hospitals of this character at the present juncture, when the care of the children is a matter of pressing urgency. That a Public Health Committee should recommend such a course is really an astonishing revelation of the way in which it interprets its duty.

### THE HOSPITAL IDEAL IN AUSTRALIA.

WE are so much imbued with the principle of the voluntary system in this country that it is difficult for most of us to envisage the general outlook and attitude of mind which are current in countries where a different method of hospital maintenance prevails. It is, therefore, interesting to read in the *Sydney Morning Herald* a letter which it published on April 4 from the Minister for Public Health. The Minister is replying to a letter signed "Citizen," in which the assertion was made that the Government discouraged private contributions to hospitals. "We welcome gladly all public subscriptions," he writes, "but, at the same time, don't believe in going cap in hand to beg. That would be just as degrading as putting up the sign over all hospital doors 'None but the very poor need apply here.' That is evidently what 'Citizen' wants, and I am not going to permit it to be done; especially as the State provides, roughly, three-fourths of the funds used by the hospitals, which to that extent are nationalised already." It is interesting to have these remarks of the Minister for Public Health, since they are unimpeachable evidence of the attitude of mind on which the Australian hospital system is built up, and the nature of which is little realised in this country.

### RECOGNITION OF AN INFIRMARY MEDICAL SUPERINTENDENT.

The Camberwell Board of Guardians have found means by which they can circumvent the decision of the Local Government Board not to sanction any extra remuneration to Dr. Keats, the medical superintendent of the infirmary, as recorded in The Hospital on June 19. The refusal of the Local Government Board was referred to their infirmary committee, whose attention was called by Dr. Keats to the extra work he was required to carry out under the Old Age Pension Act and the National Insurance Act. The committee ascertained that the Guardians were empowered to pay out of the rates reasonable expenses incurred in the preparation and collection of information required of them respecting any matter which was submitted to their management, and they therefore recommended that Dr. Keats should be paid £75 for the extra work in this connection which had fallen upon him. The proposal created a considerable amount of discussion when it came before the Camberwell Board of Guardians for consideration, but by a very large majority the committee's recommendation was carried. The Board have worked hard to get the Local Government Board to recognise the magnitude of Dr. Keats's services to the poor of the borough, and failing this have adopted their own method of recognition, which is as ingenious as it promises to be successful.

### FRENCH RED CROSS AND THE DARDANELLES.

A need has arisen for the hospital accommodation of serious cases in connection with the operations at the Dardanelles, as a result of which one of the vessels of the Messageries Maritimes, the *Charles Roux*, has been placed at the disposal of the French Red Cross, and is being fitted out and equipped as a hospital ship. The French Red Cross will be responsible for the staff of doctors and nurses, and will supply the necessary equipment and medical stores, but the coal will be furnished by the French Government. In addition to these preliminary essentials, however, there are, of course, required a variety of supplementary stores, such as linen, socks, pyjamas, to say nothing of comforts for the wounded. All such things, including bed-rests and ice-machines, will have to be supplied by private charity, and since both French and British wounded will be accommodated upon the vessel, English people have as personal an interest as the French in seeing that these imperative wants shall be supplied promptly. On the promptitude of the public, in fact, depends the early sailing of the vessel, which, generous help being forthcoming, should be ready to start in about a month's time. The London address for all gifts to be sent to is "Care of Mme. de la Panaise, French Red Cross, Knightsbridge," and marked "*Charles Roux* Fund." When it is added that the vessel is required for cases which cannot be immediately removed to base hospitals, the importance of the work which it will undertake, and the need for making provision for these serious cases, become apparent.

### THE OFFICIAL RESPIRATOR.

The announcement has already been made by the War Office that an improved type of respirator has been adopted, which is based on the recommendation of an expert committee. This, we understand, has now been issued to the troops in France, and in connection with it a general order has apparently been issued to the troops which forbids the use of any other type. The public are therefore asked not to send respirators to their soldier friends. To some extent this should affect the recent boom in respirators at home, since the brisk sale of the varied types now on the market has been due not only to the fear of Zeppelin poison bombs at home, but also, as the prohibition indicates, to the desire of parents and relations to dispatch a respirator to their relative at the Front. The adoption of an official respirator, however, suggests the question whether it would not be possible to standardise a type, for the protection of the public, in much the same way as certain fire extinguishers are singled out for official approval. For such a purpose the War Office need not officially act; sanction could be given by some, so to speak, neutral body, as is the case with the fire extinguishers mentioned above. The suggestion is made for what it may be worth, and we should be glad to have our readers' opinions on the point. Should a standardised respirator be available for the civilian population in this country?

### THE WOUNDED UP THE THAMES.

On Tuesday afternoon the Port of London Authority's steamer *Conservator* started from the Temple Pier on its first trip up the river with a passenger list of wounded. The party comprised men of both Services from King George Hospital, Stamford Street, who arrived between 1.30 and 2, the hour of starting, in brakes and ambulance wagons. The men were seen off by Mr. Edmund Owen, surgeon-in-chief, St. John Ambulance Brigade, and Mr. F. Hastings, secretary of the British Red Cross Society. On board were also Lord Devonport, chairman of the Port of London Authority, and several others. During the trip, which was timed to last about four hours, various games were played, and tea, of course, was provided. During the summer, and weather permitting, similar trips, it is hoped, will be able to be taken every day except Saturdays and Sundays. Since the starting points are also understood to include the Greenwich and Blackwall Piers, the delightful deduction can be drawn that not only men in the central institutions of London will benefit by the Port of London's hospitality. The arrangement seems to us a capital one, and from many points of view the river is preferable to the road as a highway for the wounded; and the steamer has obvious advantages over the motor for the men's enjoyment. Lord Devonport and the Port Authority are to be congratulated on their kind thought, which is likely to be very much appreciated.

### THE NEW HOSPITAL AT ENGLEFIELD GREEN AND ITS PROMOTERS.

Sir A. Markham, M.P., has lately raised many questions of public interest in the House of Commons, and, though the following is somewhat lengthy, it should be placed on record, as concerning hospital affairs. On Friday last week he asked the Under-Secretary of State for War whether it was the intention of the War Office, through the Red Cross Society, to erect a hospital of 120 beds in a field near Englefield Green, Surrey, on Crown property; whether Baron Bruno von Schroeder, Baron von Laurentz, Messrs. Gunthers, Donners, Schwartz, Nelke, and Baumanns were the promoters of this scheme; if so, would he say why Heath Lodge, Englefield, a new house recently completed belonging to Baron von Schroeder, which had accommodation for at least 120 beds and was equipped with ten bathrooms, should not be used for this purpose, seeing that the Baron was living at a large house close by; and whether, seeing that it was only at the request of the Governor of the Bank of England that Baron von Schroeder, a then German alien enemy, was naturalised after the war for financial reasons, he would now ask Baron von Schroeder, seeing that he had not been interned, to hand this new house over to the Government for a hospital. Mr. Tennant, in reply, said: "Her Royal Highness Princess Christian is organising a hospital for 120 men near Englefield Green. We have no knowledge of the other circumstances mentioned in the question." Since, however, the person who "organises" a hospital is not necessarily a "promoter," the Under-Secretary's reply hardly answers Sir A. Markham's second point.

### THE NON-PANEL MOVEMENT.

Last Saturday Dr. D. V. Greenyer, as chairman of the council, presided over the annual general meeting of the National Medical Union, and at the annual dinner the same evening recalled how the combination of non-panel medical men came into being. The genesis of the Union was recorded step by step in our columns, and it is interesting to learn from Dr. H. B. Greene's speech at the dinner that three provincial sections have been organised by Dr. E. A. Dorrell, the hon secretary, and that the influence of the Union extends to Jersey and the Isle of Man. Dr. Edwin Smith, who gave our readers an account of the non-panel movement in its early days of opposition to the Insurance Act, said that the Union was the only body which existed to preserve the traditional freedom of the profession. It should be noted that Dr. Greenyer also stated at the dinner that the Union hoped at a not very remote date to place a scheme before its members. In this, no doubt, the principles for which it stands in relation to a satisfactory medical service for necessitous persons will be given a practical shape. We welcome this promise of a constructive policy, though the times are out of joint, as we all know. The most important resolution at the annual meeting was one which authorised the Union to make public the dangers to the public health of the Insurance Acts and the injustice to doctor and patient alike of the present conditions. To this *exposé* a constructive policy on the Union's lines would give great weight, and is therefore to be welcomed. Another resolution, by the way, expressed the Union's belief in "the physical and moral value of military training for young men of all classes."

### THIS WEEK'S DRUG MARKET.

There is no falling-off in the volume of business notwithstanding the continual advances in prices. Week by week these advances continue, and it seems likely that before long some commonly used drugs that were formerly in abundant supply will become scarce almost to the point of temporary extinction. Recently the tendency to dearer rates has involved a wide range of drugs, and the occasions on which an article of medicinal produce becomes reduced in value are comparatively rare. Cod-liver oil has begun to move upwards in price again; apparently this is due to increased buying in Norway. Potassium permanganate is much dearer. Makers of corrosive sublimate and salts of mercury generally have withdrawn their quotations as a result of the further advances in the price of quicksilver. Rectified spirit is dearer, and in consequence there will be small advances in the prices of tinctures and other spirituous preparations; this advance is due to the increased cost of the production of spirit and is quite distinct from the question of recent spirit legislation. Citric acid and cream of tartar are again dearer. Essence of lemon has an upward tendency in price. Benzoic acid and benzoates are dearer. Bromides seem likely to advance in price again. The high values of salicylic acid and the salicylates are well maintained. The price of glycerine is as yet unchanged. Opium has an upward tendency in price. Preliminary reports from English medicinal herb farms seem to indicate that, given favourable weather, the crops on the whole will be good.

### TO OUR READERS.

Contributions are specially invited from any of our readers to these columns. They should deal with topical subjects and news. They must be authenticated for the information of the Editor only. The minimum payment if published is 5s. There is no hard-and-fast rule as to space, but notes of about twenty lines in length are preferred.

